# Joint probability calculation of the lateral velocity distribution in strong field ionization process

**DOI:** 10.1038/s41598-022-24168-8

**Published:** 2022-11-14

**Authors:** I. A. Ivanov, Kyung Taec Kim

**Affiliations:** 1 Center for Relativistic Laser Science, Institute for Basic Science, Gwangju, 61005 Korea; 2 Department of Physics and Photon Science, Gwangju Institute of Science and Technology, Gwangju, 61005 Korea

**Keywords:** Atomic and molecular interactions with photons, Attosecond science

## Abstract

We describe an approach to the description of the time-development of the process of strong field ionization of atoms based on the calculation of the joint probability of occurrence of two events, event B being finding atom in the ionized state after the end of the laser pulse, event A being finding a particular value of a given physical observable at a moment of time inside the laser pulse duration. As an example of such an physical observable we consider lateral velocity component of the electron’s velocity. Our approach allows us to study time-evolution of the lateral velocity distribution for the ionized electron during the interval of the laser pulse duration. We present results of such a study for the cases of target atomic systems with short range Yukawa and Coulomb interactions.

## Introduction

The conventional framework for understanding the basic features of the tunneling ionization process, known as the Strong Field Approximation (SFA), was laid out by Keldysh^1^ and developed and refined subsequently^2–9^. In this framework the tunneling regime of ionization is defined as the regime characterized by the small values of the so-called Keldysh parameter $\gamma =\omega \sqrt{2|\varepsilon _0|}/E$ (here $\omega$, *E* and $|\varepsilon _0|$ are the frequency, field strength and ionization potential of the target system expressed in atomic units).

A number of nonlinear phenomena, such as above-threshold ionization (ATI), high harmonic generation (HHG) and non-sequential double ionization (NSDI) may occur in this regime. The most essential features of these processes can often be understood with the help of the well-known simple man model (SMM)^6,10–13^, which represents electron’s motion after the ionization event occurs as entirely classical. This fact underlies a variety of highly efficient semi-classical approaches^12–19^, in which the electron ionization event is treated quantum-mechanically, with electron’s ionization occurring near the local peaks of the electric field, and the subsequent electron motion treated classically or semi-classically. These approaches include the well-known TIPIS model^12,16,20^, the quantum trajectory Monte Carlo model (QTMC)^19^, semi-classical two-step model^21^ or Coulomb quantum orbit strong-field approximation (CQSFA)^22,23^.

Despite these semi-classical methods giving often impressive agreement with more rigorous calculations based on the solution of the time-dependent Schrödinger equation (TDSE), it is not quite clear what the very notion of the ’ionization event’ means from the point of view of the conventional quantum mechanics (QM). Indeed, this notion is not easy to define even in classical physics. This problem manifests itself in the treatment of ionization in the standard QM framework in the following way. QM provides us with only a wave-function to describe evolution of the system in the laser field, and for the moments of time inside the interval of the laser pulse duration, when the wave-packet describing ionized electron has not left the atom yet, it is difficult to unambiguously single out the part of the wave-function describing ionized electron from the total wave-function of the system. At first glance such a separation of the total TDSE wave-function into the bound component and the ionized wave-packet arises quite naturally in the SFA or the Perelomov-Popov-Terent’ev (PPT) approaches^1–4,8,24^. We should note, however, that this splitting of the total wave-function in two components in these theories, although extremely useful and physically appealing, is not quite rigorous. That can been seen, for instance, by noting that this splitting is not gauge-invariant. It is this fact that is ultimately responsible for the lack of the gauge-invariance of the SFA or PPT approaches^9^. To see more clearly the origin of the problem we will recapitulate briefly, following the work^25^, the theoretical foundations of both approaches, which can done most easily by using the notion of the unitary time-evolution operator $\hat{U}(t,\tau )$ which maps state of the system at time $\tau$ to the state at time *t*. For a system with the time-dependent Hamiltonian operator $\hat{H}(t)=\hat{H}_A(t) + \hat{B}(t)$. the integral Dyson equation for the evolution operator $\hat{U}(t,\tau )$ can be written in two equivalent forms as:

1 $$\begin{aligned} \hat{U}(t,0)= & {} \hat{U}_A(t,0) -i\int \limits _0^t \hat{U}_A(t,\tau ) \hat{B}(\tau )\hat{U}(\tau ,0)\ d\tau \nonumber \\ \hat{U}(t,0)= & {} \hat{U}_A(t,0) -i\int \limits _0^t \hat{U}(t,\tau ) \hat{B}(\tau )\hat{U}_A(\tau ,0)\ d\tau \, \end{aligned}$$

where $\hat{U}_A(t,\tau )$ is the time-evolution operator describing evolution driven by the Hamiltonian operator $\hat{H}_A$. In the case of the Hamiltonian which describes atomic or molecular system interacting with the electromagnetic field: $\displaystyle \hat{H}(t)=\hat{T} + \hat{V} + \hat{H}_{\text{int}}(t)$, where $\hat{T}$ is the kinetic energy operator, $\hat{V}$- the potential energy operator, and $\hat{H}_{\text{int}}(t)$ describes the interaction of the system and the electromagnetic field, two useful partitions of the total Hamiltonian $\hat{H}(t)$ are:

2 $$\begin{aligned} \hat{H}(t)= & {} \hat{H}_{\text{atom}} + \hat{H}_{\text{int}}(t) \nonumber \\ \hat{H}(t)= & {} \hat{H}_{F}(t) + \hat{V} \ , \end{aligned}$$

where $\hat{H}_{\text{atom}} = \hat{T} + \hat{V}$ is the field-free Hamiltonian of the system, $\hat{H}_F(t)= \hat{T} + \hat{H}_{\text{int}}(t)$ is the so-called Volkov Hamiltonian. Using these two partitions in the Dyson equations (1) , one obtains two equations:

3 $$\begin{aligned} \hat{U}(t,0)= & {} \hat{U}_0(t,0) -i\int \limits _0^t \hat{U}(t,\tau ) \hat{H}_{\text{int}}(\tau )\hat{U}_0(\tau ,0)\ d\tau \nonumber \\ \hat{U}(t,0)= & {} \hat{U}_F(t,0) -i\int \limits _0^t \hat{U}_F(t,\tau ) \hat{V} \hat{U}(\tau ,0)\ d\tau \ , \end{aligned}$$

where $\displaystyle \hat{U}_0(t,\tau ) = \exp {\left\{ -i\hat{H}_{\text{atom}}(t-\tau )\right\} }$ is the evolution operator describing evolution driven by the time independent field-free Hamiltonian $\hat{H}_{\text{atom}}$ and $\hat{U}_F(t,\tau )$ is the so-called Volkov evolution operator with a simple known analytical form^9^ which describes quantum evolution driven by the Volkov Hamiltonian $\hat{H}_F(t)$.

Equations (3) are exact but are not very useful, being just as difficult to solve as the original TDSE with which we started. The widely used and important SFA and PPT approximations are obtained if we substitute $\hat{U}_F(t,\tau )$ for $\hat{U}(t,\tau )$ under the integral in the first Dyson equation (1) and $\hat{U}_0(\tau ,0)$ for $\hat{U}(\tau ,0)$ in the second equation (1):

4 $$\begin{aligned} \hat{U}_{SFA}(t,0)= & {} \hat{U}_0(t,0) -i\int \limits _0^t \hat{U}_F(t,\tau ) \hat{H}_{\text{int}}(\tau )\hat{U}_0(\tau ,0)\ d\tau \nonumber \\ \hat{U}_{PPT}(t,0)= & {} \hat{U}_F(t,0) -i\int \limits _0^t \hat{U}_F(t,\tau ) \hat{V} \hat{U}_0(\tau ,0)\ d\tau \ . \end{aligned}$$

If we use the expression for the evolution operator in the first equation (4) to find the wave-function at the moment of time $t=T_1$ at the end of the laser pulse for a system which was initially in the field free state $\phi _0$, we obtain an approximate wave-function: $\Psi _{SFA}(T_1)= \hat{U}_{SFA}(T_1,0)\phi _0$. Projecting this wave-function on a plane-wave state $|{{\varvec{k}}}\rangle$ and dropping the term $\langle {{\varvec{k}}}|\hat{U}_0(T_1,0)|\phi _0\rangle$, one obtains the well-known expression for the ionization amplitude used by Keldysh^1^, provided we use the length form for the interaction Hamiltonian $\hat{H}_{\text{int}}(t)$. If, instead, we use the velocity gauge to describe field-atom interaction, we obtain the expression for the ionization amplitude used in the well-known Strong Field Approximation (SFA) theory^2,3^. Using, on the other hand, the approximate evolution operator in the second equation (4) to evaluate the wave-function at the moment of time $t=T_1$ at the end of the laser pulse for a system which was initially in the field free state $\phi _0$, we obtain an approximate wave-function $\Psi _{PPT}(T_1)= \hat{U}_{PPT}(T_1,0)\phi _0$. Projecting this wave-function on a plane-wave state $|{{\varvec{k}}}\rangle$ and dropping the term $\langle {{\varvec{k}}}|\hat{U}_F(T_1,0)|\phi _0\rangle$ which does not contribute to the probability current, one obtains the expression for the ionization amplitude used in the Perelomov-Popov-Terentiev (PPT) theory^4^.

We see, thus, that both in the PPT and SFA approaches we obtain expressions for the ionization amplitudes by omitting certain terms from the total wave-function. These terms, however, are different in the two approaches and, moreover, it is this omitting that breaks the gauge invariance which is of course present in the complete theory. Under a gauge transformation only the total wave-function of a system is transformed in a way ensuring gauge invariance of the final results for the observables. The parts of the wave-function obtained by splitting it into different components will not, in general, possess this property. One needs some, therefore, additional theoretical ingredients in the theory, which may allow to define the notion of the ionized part of the wave-function. An example of such a theoretical development is provided by the well-known back-propagation technique^26–30^. This procedure allows to single out only ionized electron and to follow its prehistory back in time by constructing the ionized wave-packet from the part of the coordinate wave-function localized far from the nucleus for times long after the end of the driving pulse.

In the present work we describe another procedure allowing to achieve such a separation of the total wave-function describing the evolution of an atom in the laser field into the ionized and non-ionized components. We will use the notion of the conditional and joint probabilities for this purpose. In the Section Theory below we describe our theoretical procedure. We will illustrate this procedure by applying it to a study of the evolution of the lateral velocity distribution (i.e. distribution of the velocity components in the directions orthogonal to the polarization vector of the driving field) of ionized electrons for times inside the laser pulse duration. These applications are described in the Section Results below. We conclude in the Section Conclusion making a brief summary of the results and future perspectives. Atomic units (a.u.) are used throughout the paper.

## Theory

### Joint probability in Quantum Mechanics

We begin by presenting a short summary of the theory of the conditional and joint probabilities in QM following work^31^. Suppose we have a system described by the state vector $|\Psi \rangle$, and two observables with the corresponding Hermitian operators $\hat{A}$ and $\hat{B}$. The probability to find the system upon measurement in an eigenstate of $\hat{B}$ belonging to a spectral interval $\Delta _B$ of the operator $\hat{B}$, can be computed, as is well known, as follows:

5 $$\begin{aligned} P(\Delta _B)= \langle \Psi |\hat{Q}(\Delta _B)|\Psi \rangle \ , \end{aligned}$$

where $\hat{Q}(\Delta _B)$ is the Hermitian projection operator which can be expressed in terms of the eigenstates $|\lambda \rangle$ of the operator $\hat{B}$ as: . Analogous formula, where we will have to use a projection operator $\hat{Q}(\Delta _A)$, which can be similarly expressed in terms of the eigenstates of the operator $\hat{A}$, can be written, of course, for the probability $P(\Delta _A)$ to find the system in an eigenstate of $\hat{A}$ belonging to a spectral interval $\Delta _A$ of the operator $\hat{A}$.

Suppose now that we are interested in the joint probability $P(\Delta _A  \&  \Delta _B)$ of finding observable *A* in the spectral interval $\Delta _A$, and observable *B* in the spectral interval $\Delta _B$. One might try to define such a joint probability by an expression:

6 $$ \begin{aligned} P(\Delta _A  \&  \Delta _B) = \langle \Psi |\hat{Q}(\Delta _A)\hat{Q}(\Delta _B)|\Psi \rangle \ . \end{aligned}$$

This definition would make perfect sense if the quantum projection operators $\hat{Q}(\Delta _A)$ and $\hat{Q}(\Delta _B)$ were classical quantities or at least quantum commuting operators. Unfortunately, this is not necessarily always the case. If the operators $\hat{A}$ and $\hat{B}$ do not commute, the corresponding projection operators $\hat{Q}(\Delta _A)$ and $\hat{Q}(\Delta _B)$ do not commute either, the operator product $\hat{Q}(\Delta _A)\hat{Q}(\Delta _B)$ in Eq. (6) becomes non-Hermitian and the joint probability defined by Eq. (6) is generally complex-valued. This is, of course, another manifestation of the well-known difficulty that one encounters when trying to assign a meaning to the joint probability distributions of the observables described by non-commuting operators using the quasi-probability distributions, such as Wigner and Husimi distributions^32–35^ which may not necessarily be strictly positive. With Eq. (6) we have the same problem in another disguise, it can give, as we see, complex-valued joint probability distributions. It can be argued^31,36,37^ that such quasi-probability distributions, which are not strictly positive, can be incorporated in the framework of the QM, and that they appear naturally in the description of the classically forbidden processes such as the tunneling process^36^. Negative probabilities are, however, difficult to interpret, and many physicists are somewhat reluctant in accepting such a notion.

It is the tunneling process due to atomic ionization in the external electric field that interests us in the present work. We will see below that in some circumstances Eq. (6) gives us joint probability distributions which are perfectly legitimate in an ordinary probabilistic sense, and which may provide useful information about the development of the tunneling ionization process.

### Joint probabilities for the strong field ionization process

We will be studying below ionization of a single-electron atom in a strong electromagnetic field. The evolution of the system in time is described by the three-dimensional TDSE:

7 $$\begin{aligned} i {\partial \Psi ({{\varvec{r}}},t) \over \partial t}= \left( \hat{H}_{\text{atom}} + \hat{H}_{\text{int}}(t)\right) \Psi ({{\varvec{r}}},t) \ , \end{aligned}$$

with $\displaystyle H_{\text{atom}}= {\hat{{\varvec{p}}}^2\over 2}+V(r)$- field free atomic Hamiltonian, and $\hat{H}_{\text{int}}(t)$- interaction Hamiltonian describing atom-field interaction. We use the length gauge for the latter operator:

8 $$\begin{aligned} \hat{H}_{\text{int}}({{\varvec{r}}},t) = {{\varvec{r}}}\cdot {{\varvec{E}}}(t) \, \end{aligned}$$

where ${{\varvec{E}}}(t)$ is electric field of the pulse. We assume that pulse is linearly polarized along *z*-axis. The pulse is defined in terms of the vector potential: $\displaystyle {\varvec{E}}(t)=-{\partial {{\varvec{A}}}(t)\over \partial t}$, where:

9 $$\begin{aligned} {{\varvec{A}}}(t)= -{\hat{{\varvec{e}}}_z}{E_0\over \omega }\sin ^2{\left( {\pi t\over T_1}\right) }\sin {\omega t} \ , \end{aligned}$$

where $T_1$ is the total pulse duration, which we choose to be one optical cycle (o.c.): $T_1=2\pi /\omega$, for the majority of the calculations reported below. We use a single cycle pulse for purely computational reasons as the calculations become rather time-consuming for longer pulses, our approach can be generally applied for long pulses as well. We will present below results for different values of the pulse base frequency $\omega$, peak field strength $E_0$ and different atomic potentials *V*(*r*).

To apply Eq. (6) and the notion of the joint probability distribution in QM to the process of the tunneling ionization we should first specify the operators $\hat{A}$ and $\hat{B}$ in this equation. We note first that it is by no means necessary that both projective measurements in Eq. (6) are performed at the same moment of time. We may well assume that one of the projective measurements (let’s say the measurement described by the projection operator $\hat{Q}(\Delta _B)$) is performed at the time $t_2$, while the measurement described by the operator $\hat{Q}(\Delta _A)$ is performed at the time $t_1$, with $t_2>t_1$. To modify Eq. (6) accordingly it is convenient to use the Heisenberg picture of the QM, in which the state vector $\Psi =\phi _0$ is independent of time (here $\phi _0$ is the initial state of the system), while the projection operators $\hat{Q}(\Delta _A)$ and $\hat{Q}(\Delta _B)$ evolve in time. With the use of the Heisenberg picture the generalization of the Eq. (6) for the case of different times is straightforward:

10 $$ \begin{aligned} P(\Delta _A(t_1)  \&  \Delta _B(t_2)) = \langle \phi _0|\hat{Q}^H(\Delta _A,t_1)\hat{Q}^H(\Delta _B,t_2)|\phi _0\rangle \ , \end{aligned}$$

with

11 $$\begin{aligned} \hat{Q}^H(\Delta _A,t_1)= & {} \hat{U}(0,t_1) \hat{Q}(\Delta _A)\hat{U}(t_1,0) \nonumber \\ \hat{Q}^H(\Delta _B,t_2)= & {} \hat{U}(0,t_2) \hat{Q}(\Delta _B)\hat{U}(t_2,0) \ , \end{aligned}$$

where $\hat{U}(t,0)$ is the evolution operator, driving quantum evolution of the system, and $|\phi _0\rangle$ is the initial atomic state (which we assume to be the ground state of the field-free atomic Hamiltonian $H_{\text{atom}}$.

Definition Eq. (10) of the joint probability distribution is quite similar to the definition of the two-time correlation function^38–41^, describing correlations between two observables with corresponding quantum mechanical operators $\hat{Q}(\Delta _A)$ and $\hat{Q}(\Delta _B)$ at times $t_1$ and $t_2$, respectively. Correlation functions can be employed^42,43^ to study correlations between different observables in the strong field ionization process. Such a study may provide useful information about presence or absence of the correlations between observables at different moments of time. On the other hand, the two-time correlation functions, being generally complex-valued objects, do not have direct physical interpretation. The approach we describe in the present work differs from the approach based on the two-time correlation functions analysis in one important aspect. We assume that the quantum operators $\hat{Q}^H(\Delta _A,t_1)$ and $\hat{Q}^H(\Delta _B,t_2)$ in Eq. (10) are are not arbitrary operators, but quantum mechanical projection operators. This implies that both $\hat{Q}^H(\Delta _A,t_1)$ and $\hat{Q}^H(\Delta _B,t_2)$ are positive-definite Hermitian operators. Moreover, if $\hat{Q}^H(\Delta _A,t_1)$ and $\hat{Q}^H(\Delta _B,t_2)$ are commuting operators, the operator product $\hat{P}=\hat{Q}^H(\Delta _A,t_1)\hat{Q}^H(\Delta _B,t_2)$ in Eq. (10) is again a positive definite Hermitian operator with positive real expectation value. It is easy to see, in fact, that $\hat{P}$ is then a projection operator, satisfying $\hat{P}^2=\hat{P}$. If $\hat{Q}^H(\Delta _A,t_1)$ and $\hat{Q}^H(\Delta _B,t_2)$ commute we can, therefore, assign direct physical meaning of the joint probability to the quantity defined by the Eq. (10). We will now specify the observables *A* and *B* and the projection operators $\hat{Q}(\Delta _A)$ and $\hat{Q}(\Delta _B)$ in Eq. (11).

#### Choice of the observable *B*

We will assume from now on that in Eq. (10) the later moment of time $t_2=T_1$, the moment of time when the laser pulse (9) is switched off. For the projection operator $\hat{Q}(\Delta _B)$ we will use the projection operator projecting the state vector on the continuous part of the energy spectrum of the atomic Hamiltonian $H_{\text{atom}}$,and by the spectral interval $\Delta _B$ we will understand the whole range of positive electron energies of the continuous spectrum of the field-free atomic Hamiltonian. In other words, $Q(\Delta _B) =\hat{Q}_{c}$, where $\hat{Q}_c$ is the projection operator on the continuous spectrum of the field-free atomic Hamiltonian, so that for any state vector $|\Psi \rangle$:

12 $$\begin{aligned} \hat{Q}_c |\Psi \rangle = |\Psi \rangle - \sum \limits _{\begin{array}{c} {bound}\\ {states} \end{array}}\langle \phi _b|\Psi \rangle |\phi _b\rangle \ , \end{aligned}$$

where the sum on the r.h.s includes all bound states of the field-free atomic Hamiltonian $H_{\text{atom}}$. It is worth discussing briefly what we have achieved by choosing this particular form of the projection operator $\hat{Q}(\Delta _B)$. Observable *B* with this choice of the projection operator provides basically the answer to the question: will the electron be ionized or not, it might be pictured as an observable having the value one if electron is ionized, and zero otherwise. The joint probability distribution (10) (provided it gives a legitimate probability distribution, we will touch on this question later) gives us therefore a joint distribution of the observable *A* at the time $t_1$ inside the laser pulse duration upon the condition that electron will be ionized in the future. By using the joint probability approach and our choice of the observable *B* we are able, therefore, to separate ionized and non-ionized electrons. As we noted in the Introduction such a separation is by no means trivial and requires some additional theoretical notions, such as the notions based on different ionization criteria employed in the back-propagation technique^26–30^. We can look at the Eq. (10) as yet another way to achieve this separation and study prehistory of the ionized electron based on the notion of joint probability distribution and the choice of the observable *B* we made above.

#### Choice of the observable *A*

As far as the observable *A* in Eq. (10) is concerned, we can choose, in principle, any observable which will provide useful information about the ionization process. Our only limitation is that the choice of *A* must be such that the joint distribution (10) be at least approximately legitimate probability distribution. What it means in practice is that we must choose *A* so that the imaginary part of the distribution (10) be small comparing to its real part for all values of *A* of interest, i.e. for the bulk of the distribution where the joint probability has non-negligible values. If this condition is satisfied, the projection operators in Eq. (10) will (approximately) commute, and Eq. (10) will automatically give a joint distribution which will be positive at least for the bulk of the distribution. We will, of course, still have non-physical negative probabilities on the edges of the distribution, where the joint probability is small. That is inevitable unless the projection operators $\hat{Q}(\Delta _A,t_1)$ and $\hat{Q}(\Delta _B,t_2)$ in Eq. (10) strictly commute, but that is presumably not very important.

That was the strategy we pursued in choosing the observable *A*. It turns out that, while it is not generally possible to choose a physically interesting observable *A* so that imaginary part of the joint distribution (10) be always small, it is possible to achieve this goal for at least some values of time $t_1$ inside the pulse. That can still provide us with valuable information about development of the ionization process and evolution of the observables characterizing ionized electrons inside the interval of the pulse duration .

An observable *A* which we will study below in detail is the lateral component of electron’s velocity, i.e component of the velocity perpendicular to the polarization vector of the laser pulse ($z-$direction for the geometry we use in Eq. (9)). It will be convenient in the following to use a cylindrical coordinate system $(\rho ,\phi ,z)$ in the vector space of electron’s velocities ${{\varvec{v}}}$ with the longitudinal axis along the $z-$ direction. Let us consider a set of regions $\Omega _k$ of the electron’s velocity space, where integer $k=0,1\ldots$, such that the components of the vector ${{\varvec{v}}}$ in the cylindrical coordinate system we introduced above satisfy for any integer *k*: $\displaystyle \Omega _k= ( kd \le v_\rho< (k+1)d; -\infty< v_z < +\infty )$. In other words, $\Omega _k$ is the space between two cylinders of infinite height and radii of *kd* and $(k+1)d$. In the calculations below we use $d=0.1$ a.u. Parameter *d* affects the overall ’resolution’ in the velocity space which we may hope to achieve in the framework of our approach. We should choose is so as to not to loose any important fine details of the lateral velocity distributions. We will justify the choice of the value $d=0.1$ a.u. that we made below. Let $\chi _{\Omega _k}({{\varvec{v}}})$ be the characteristic function of the region $\Omega _k$ (i.e. $\chi _{\Omega _k}({{\varvec{v}}})=1$ for ${{\varvec{v}}}$ inside $\Omega _k$, $\chi _{\Omega _k}({{\varvec{v}}})=0$ otherwise). We define now the action of the projection operator $\hat{Q}_k$ on a state vector $|\Psi \rangle$ as follows. We first perform Fourier transform of the coordinate wave-function $\Psi ({{\varvec{r}}})$ obtaining momentum space wave-function $\tilde{\Psi }({{\varvec{v}}})$. We define now $\hat{Q}_k|\Psi \rangle$ as an inverse Fourier transform of $\tilde{\Psi }({{\varvec{v}}})\chi _{\Omega _k}({{\varvec{v}}})$. With this definition $\langle \Psi |\hat{Q}_k|\Psi \rangle$ is clearly the probability to detect electron’s velocity in the region $\Omega _k$ of the velocity space we defined above. The regions $\Omega _k$ with $k=0,1\ldots$ in the velocity space cover all the space and do not intersect, they satisfy, therefore, $\hat{Q}_i\hat{Q}_j=\delta ^i_j \hat{Q}_i$. We now identify $\hat{Q}_k$ with $\hat{Q}(\Delta _A)$ where, for every integer *k* it is understood that spectral region $\Delta _A$ coincides with the region $\Omega _k$ in the velocity space. In physical terms, distribution (10) in which we substitute projection operator $\hat{Q}_k$ for observable *A* gives us a probability of finding electron’s velocity anywhere in the region $\Omega _k$ of the velocity space at the moment of time $t_1$ provided that electron is found to be ionized at the end of the laser pulse.

### Calculation of the joint probability

Following the discussion presented in the two preceding subsections, we rewrite the formula (10) for the joint probability using more compact notation:

13 $$ \begin{aligned} P(\Omega _i(t)  \&  Q_c(T_1))= & {} \langle \phi _0|\hat{Q}_i^H(t)\hat{Q}_c^H(T_1)|\phi _0\rangle \nonumber \\= & {} \langle \Psi (t)|\hat{Q}_i \hat{U}(t,T_1)\hat{Q}_c|\Psi (T_1)\rangle \ , \end{aligned}$$

where in the first line we used the Heisenberg representation for the projection operators (as in Eq. (10)), while to derive the second line we used the transformation equations (11) and the property $|\Psi (t)\rangle = \hat{U}(t,0)|\phi _0\rangle$ of the evolution operator, so that $|\Psi (t)\rangle$ is the solution of the TDSE obtained at the moment *t* from the initial (ground) atomic state $|\phi _0\rangle$. It is the quantity on the r.h.s of the second line of Eq. (13) that was actually computed in our calculations. We did it as follows. The TDSE (7) is propagated forward in time on the interval of the laser pulse duration $(0,T_1)$ starting with an initial atomic state $|\phi _0\rangle$, thus obtaining the state vector $|\Psi (T_1)\rangle$ describing atomic system at the end of the laser pulse. Next, we apply the operator $\hat{Q}_c$ to the vector $|\Psi (T_1)\rangle$ and propagate back in time both the resulting vector and the original vector $|\Psi (T_1)\rangle$, obtaining two time-dependent state-vectors: $|\Psi _1(t)\rangle = \hat{U}(t,T_1)\hat{Q}_c|\Psi (T_1)\rangle$ and $|\Psi (t)\rangle = \hat{U}(t,T_1)|\Psi (T_1)\rangle$. For any given moment *t* inside the interval of the pulse duration we can now compute the joint probability defined by the Eq. (13) as a matrix element $\langle \hat{Q}_i \Psi (t)|\Psi _1(t)\rangle$ (we used here Hermicity of the operator $\hat{Q}_i$). This calculation requires multiple solutions of the TDSE for different regions $\Omega _k$ defining the projection operators $\hat{Q}_k$. The 3D TDSE was solved numerically using the procedure we tested and described in detail in^44–46^. The procedure relies on representing the coordinate wave-function as a series of spherical harmonics with quantization axis along the pulse polarization direction. Spherical harmonics with orders up to $L_{\text{max}}=70$ were used. The radial variable is treated by discretizing the TDSE on a grid with the step-size $\delta r=0.05$ a.u. in a box of the size $R_{\text{max}}=200$ a.u. Necessary checks were performed to ensure that for these values of the parameters $L_{\text{max}}$ and $R_{\text{max}}$ convergence of the calculations has been achieved. The solution of the 3D TDSE was propagated both forward and backward in time using the matrix iteration method^47^. We did calculations for two atomic systems: system with the field-free dynamics governed by the short range (SR) Yukawa potential $\displaystyle V(r)=-{1.9083e^{-r}\over r}$ and the hydrogen atom with Coulomb potential $\displaystyle V(r)=-{1\over r}$. Both these systems have the same ionization potential $I_p=0.5$ a.u. and we use their ground *s*-states as initial states $|\phi _0\rangle$ in the calculations below.

The joint probability distribution in Eq. (13) depends on the time moment *t* and the region $\Omega _k$ in the velocity space. We remind that this region was defined above as the volume in the velocity space between two cylinders of radii *kd* and $(k+1)d$ where $k=0,1,\ldots$: $\displaystyle \Omega _k= ( kd \le v_\rho< (k+1)d; -\infty< v_z < +\infty )$, and we used $d=0.1$ a.u.

By introducing a variable $v_{\perp }= (k+1/2)d$ the joint probability distribution in Eq. (13) can be considered as a (discretized) function of the lateral velocity $v_{\perp }$ and time *t* computed on a lateral velocity grid $v_{\perp }= (k+1/2)d$, $k=0,1,\ldots$. We will use this fact to simplify the notation yet a bit more. We define now a function:

14 $$  \begin{aligned} G(v_{\perp },t)= {\Im ( P(\Omega _k(t)  \&  Q_c(T_1)))^2 \over |P(\Omega _k(t)  \&  Q_c(T_1))|^2} \ , \end{aligned}$$

which tells whether the imaginary part of the joint probability can be considered as small for a given moment of time *t*. The choice of the particular form of the function $G(v_{\perp },t)$ is not unique, of course, and even is not very important. We only need a convenient measure allowing us to gauge the relative magnitudes of the real and imaginary parts of the joint probability defined in Eq. (13) and to judge to what extent the joint probability is a real quantity. The function defined in Eq. (14) is just the simplest choice allowing to achieve this purpose.

As we discussed above, in general, we cannot expect that for a given *t* $G(v_{\perp },t)$ vanishes for all $v_{\perp }$ altogether. We can, however, have $G(v_{\perp },t)$ small for the bulk of the $v_{\perp }$-distribution, i.e., in the region of lateral velocities where Eq. (13) has non-negligible values. In such situations we can use Eq. (13) to define a physically legitimate probability distribution for lateral velocities $v_{\perp }$. Our strategy, therefore, is to look at the regions in the $(t,v_{\perp })$-plane where we have $G(v_{\perp },t)\ll 1$. If such regions in the $(t,v_{\perp })$-plane can be found for time *t* inside the laser pulse duration we will obtain a means of calculating a physically sensible probability distribution for lateral velocities $v_{\perp }$. We can compare the distributions thus obtained with the theoretical predictions obtained in the framework of the SFA. The well-known SFA expression for the lateral velocity distribution reads^8,9^:

15 $$\begin{aligned} P^{SFA}(v_{\perp })= A \exp \left\{ -{(2I)^{1\over 2}v_{\perp }^2\over E_0}\right\} \ , \end{aligned}$$

where *I* is the ionization potential, $E_0$- electric field strength, and $v_{\perp }^2= v_x^2+ v_y^2$ for the geometry we employ. We do not specify the constant *A* in this expression as we will be interested below in the distribution shape which is described by the exponential function. Expression (15) gives us the final velocity distribution at the detector when the laser pulse is gone, but it can also be used as a plausible expression for the lateral velocity distribution within interval of the pulse duration, as is done e.g., in the semi-classical simulations^12,16,20,21^. The rational behind that is that a linearly polarized electric field does not affect the distribution in the lateral direction during the electron’s motion subsequent to the ionization event. The width of the lateral velocity SFA distribution (15) can, therefore, be used as a guide in choosing the value for the parameter *d* we used to foliate the velocity space. For the field strengths of the order of $E_0\approx 0.07$ we are interested in, the full width at half maximum (FWHM) of the distribution (15) is approximately 0.45 a.u., so our choice of $d=0.1$ a.u. allows us to foliate the velocity space into the layers thin enough so as not to loose important details of the velocity distribution. The accuracy could be increased by using a smaller value of the parameter *d* at the expense of additional computing time, but as we shall see below, with the foliation parameter $d=0.1$ a.u. the lateral velocity distribution obtained in the framework of our approach agrees pretty well with the SFA expression (15) for the short-range interaction, confirming the overall consistence and accuracy of the procedure.

To be able to compare the SFA distribution (15) to the joint distribution (13) we should take into account the fact that (13) refers to the joint probability of finding electron’s velocity in the region $\Omega _k$: $\displaystyle \Omega _k= ( kd \le v_\rho< (k+1)d; -\infty< v_z < +\infty )$ between the two cylindrical surfaces, while expression Eq. (15) refers to the Cartesian volume element in the velocity space. To take into account this difference of the volume elements we can observe that the volume $\Omega _k$ is proportional to $v_{k,\perp }$, where $v_{k,\perp }= (k+1/2)d$. We must therefore divide the joint probability in Eq. (13) by $v_{k,\perp }$ obtaining the distribution:

16 $$ \begin{aligned} P(v_{\perp },t)= {P(\Omega _k(t)  \&  Q_c(T_1)) \over v_{k,\perp } } \ , \end{aligned}$$

which refers to the Cartesian volume element in the velocity space and can, therefore, be compared with the results predicted by the SFA formula (15). We will perform such a comparison below, by fitting the results we will obtain using Eq. (16) with analytical formulas. We will employ two types of fit, one for the SR Yukawa potential and another for Coulomb potential. The SR interactions fall into the domain of the validity of the standard SFA^8,9^. For the SR interaction, therefore, we will fit our results using the fitting expression based on the SFA formula (15):

17 $$\begin{aligned} P^{SR}(v_{\perp })= A \exp \left\{ -{v_{\perp }^2\over \beta }\right\} \ , \end{aligned}$$

where *A* and $\beta$ are used as fitting parameters. Parametrization in terms of the parameter $\beta$ is convenient as for both Yukawa and Coulomb systems we have $I=0.5$ a.u. Therefore, for the lateral velocity distributions having shapes similar to the ones predicted by the SFA formula (15), parameter $\beta$ should be approximately equal to the peak field strength $E_0$ of the laser pulse.

For the Coulomb potential situation is a bit more complex. Due to the well-known effect of the Coulomb focusing^48^, lateral velocity distribution acquires a cusp at zero transverse velocity. Such a cusp-like structure can be described using an expression^16,44,49^.

18 $$\begin{aligned} P^{C}(v_{\perp })= A \exp \left\{ -{|v_{\perp }|^{\alpha }\over \beta }\right\} \ , \end{aligned}$$

where *A*, $\alpha$ and $\beta$ are used as fitting parameters. Depending on the value of the non-integer parameter $\alpha$ the distribution function in (18) exhibits at $v_{\perp }=0$ a discontinuity in first or higher order derivatives, thus serving as a model of the cusp-like behavior.

The lateral velocity distributions that we compute using Eq. (16) pertain to the moments of time insider the interval of the pulse duration. We will compare them also to the asymptotic (obtained in the limit of large times) velocity distributions $P^{as}(v_{\perp })$, which we compute using the standard prescription, by projecting the wave-function at the end of the pulse on the scattering states of the field-free atomic Hamiltonian (with ingoing boundary conditions) :

19 $$\begin{aligned} P^{as}(v_{\perp }) = \int |\langle |\phi ^-_{{\varvec{v}}}|\Psi (T_1)\rangle |^2\ dv_z \ . \end{aligned}$$

Such a comparison is of interest, especially for the Coulomb potential, as it shows how cusp in the lateral velocity distribution develops in time. It is known that the Coulomb atomic system needs to evolve for some time for the cusp to develop^50^. Comparing asymptotic lateral velocity distribution (19) to the distributions (16) we will be able to have a glimpse at how this development of the cusp actually occurs.

## Results and discussion

### Yukawa potential

**Figure 1:**
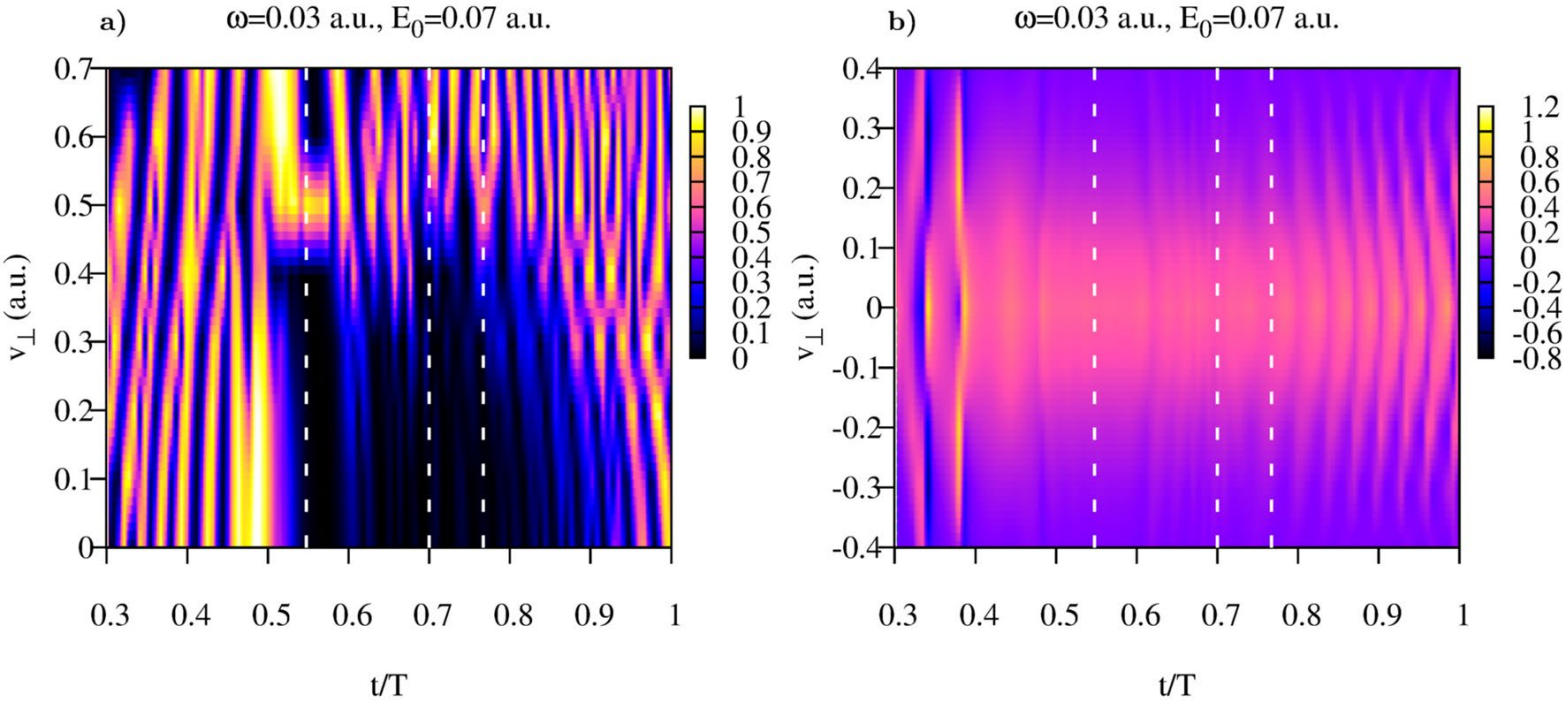
Yukawa potential. (**a**): Function $G(v_{\perp },t)$ (**b**): Distribution $P(v_{\perp },t)$. Dotted white lines show the cuts at time-values $t=0.548 T$, $t=0.7 T$, and $t=0.767 T$ for which the lateral velocity distributions shown in Figs. 2 and 3 were calculated.

We begin by presenting the results we obtain for the Yukawa potential and field parameters $\omega =0.03$ a.u., $E_0=0.07$, which places us relatively deep in the tunneling regime. In the framework of the strategy we outlined above, we will analyze first the function defined in Eq. (14) which will tell us at what particular (if any) times inside the laser pulse the notion of the joint probability can be sensibly used. This function is shown in Fig. 1a. The plot shows a rather complicated pattern, important for us are the ”good” regions in the $(t,v_{\perp })$-plane, where $G(t,v_{\perp })$ is small. We need, of course, some quantitative criterion of ”smallness”. As such, we choose the condition $G(t,v_{\perp })\le 0.1$, which, as we will try to show, is sufficiently small threshold value for the notion of the joint probability distribution to make sense. One can see from Fig. 1 that there are areas (shown in black) for times inside the laser pulse duration, where this criterion is satisfied for the large enough intervals of lateral velocities. In these areas, as we argued above, the joint probability distribution is a sensible notion, and we can compute the lateral velocity distributions by taking cuts of the Cartesian distribution $P(v_{\perp },t)$ we defined above in Eq. (16) along the lines $t={\text{const}}$.

**Figure 2:**
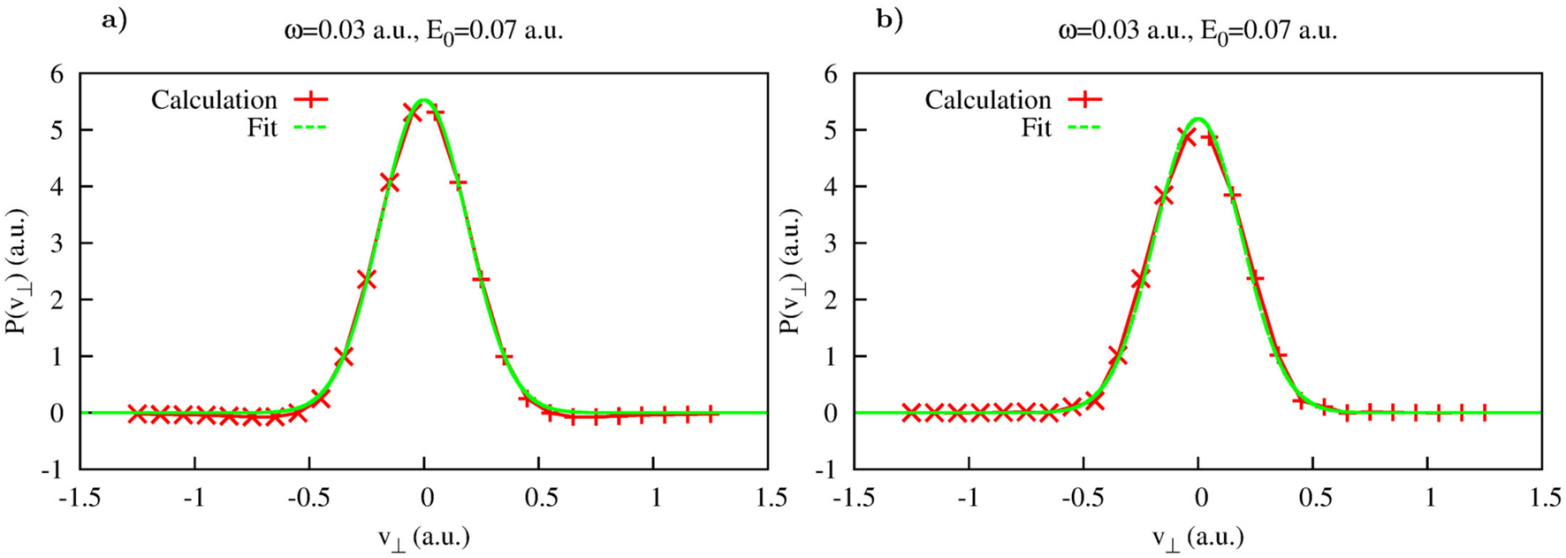
Yukawa potential. (**a**): Results of the fit of distributions computed according to Eq. (16) using Eq. (17) as a fitting anzats. (a): $t=0.548T$. (**b**): $t=0.7T$.

**Figure 3:**
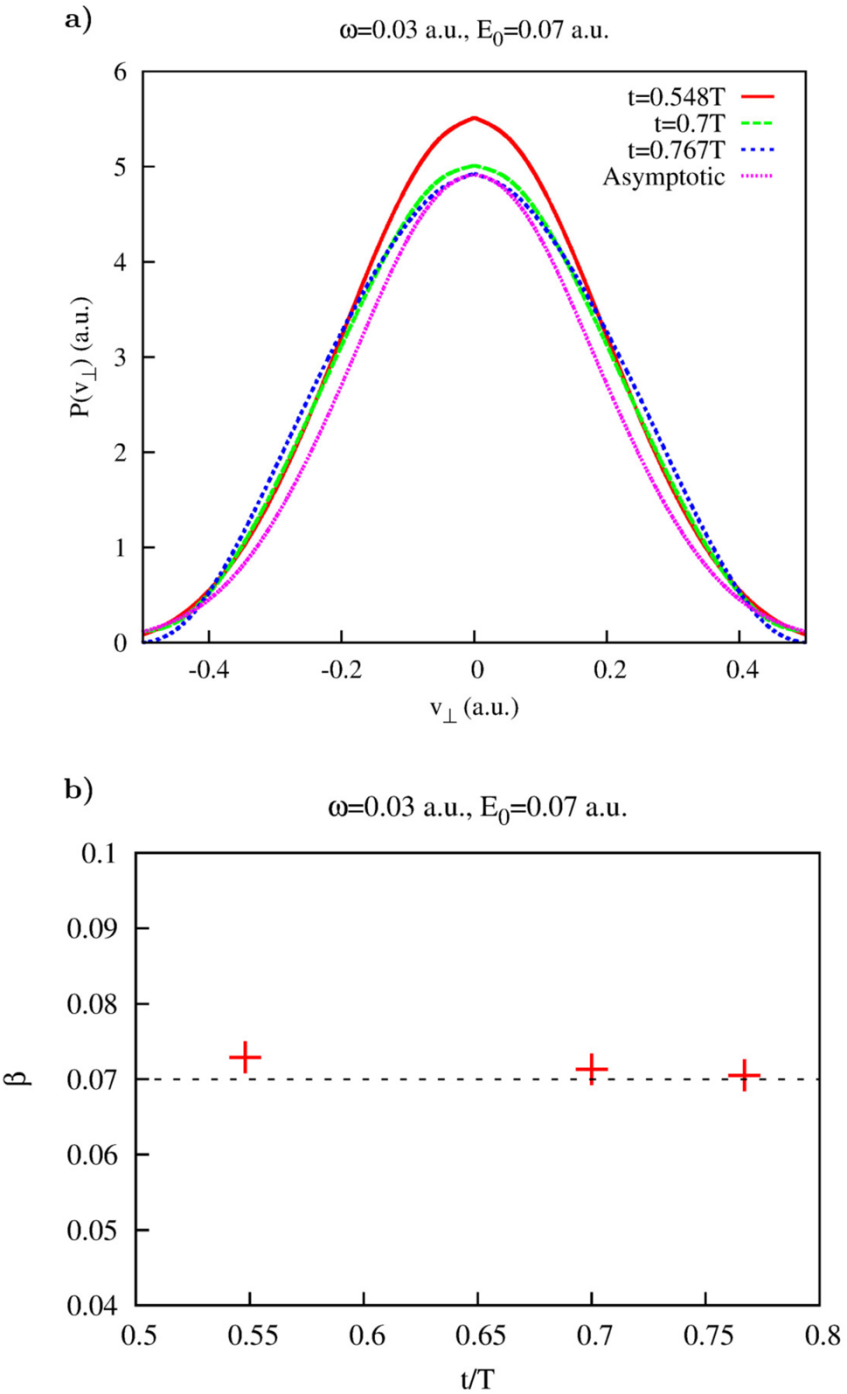
Yukawa potential. (**a**): Lateral distributions obtained using Eq. (16) for different moments of time inside the pulse duration (also shown as white dash lines in Fig. 1). Shown also is the asymptotic distribution obtained using Eq. (19). (**b**): Parameter $\beta$ of the fit as a function of time inside the interval of the laser pulse duration.

The distribution $P(v_{\perp },t)$ is shown in Fig. 1b, and its cuts $P(v_{\perp },t_i)$ taken along the lines $t=0.548 T$ and $t=0.7 T$ are shown in Fig. 2. As an inspection of the Fig. 1 shows, for both these cuts the areas where $G(t,v_{\perp })$ is small extend sufficiently far in $v_{\perp }$-direction so that Cartesian lateral velocity distributions (16) could be computed for the intervals of $v_{\perp }$ containing the bulk of the velocity distribution. The results shown in Fig. 2 for the two moments of time inside the laser pulse duration show very nice agreement with the Gaussian fit (17) based on the SFA expression (15). The prediction given by the SFA for the value of the parameter $\beta$ in Eq. (17) is $\beta =0.07$ a.u. for the field strength $E_0$ we use. The values we obtain for $\beta$ by fitting the computed lateral velocity distributions are: $\beta =0.0729$ a.u. for $t=0.548T$ and $\beta =0.0710$ a.u. for $t=0.7T$. In Fig. 3 we show evolution of the Cartesian lateral velocity distributions for the three values of time inside the interval of the laser pulse duration (indicated by the dotted white lines in Fig. 1) and compare these distributions to the asymptotic distribution $P^{as}(v_{\perp })$ defined in Eq. (19). As one can see from Fig. 3a, all these distributions, both the ones computed for times inside the interval of the pulse duration and the asymptotic distribution (19) closely agree and shape of the distributions evolves little with time. This fact is illustrated also in Fig. 3b, where we show evolution of the parameter $\beta$ in time. The pattern of the evolution we see in Fig. 3b agrees completely with the picture provided by the SFA, where electron emerges into the continuum at the moment of the maximum field strength, with the lateral velocity distribution given by Eq. (15), which does not change subsequently. This latter fact is due to the short range character of the potential which is assumed in the SFA, and the fact that in the absence of any long range forces the electric field of the pulse cannot affect the lateral components of electron’s velocity.

### Coulomb potential

**Figure 4:**
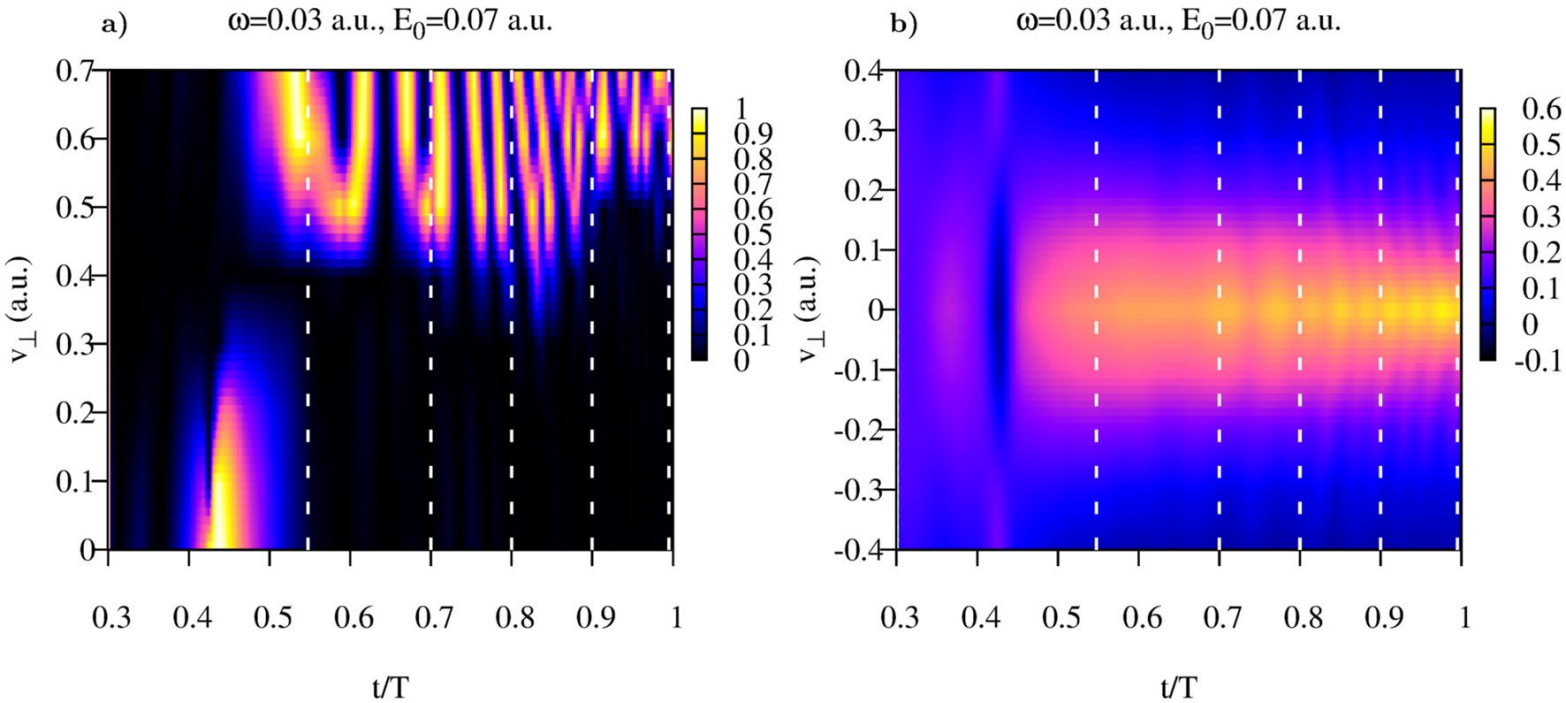
Coulomb potential. (**a**): Function $G(v_{\perp },t)$ (**b**): Distribution $P(v_{\perp },t)$.

Results that we obtain for the Coulomb potential are somewhat different. This is to be expected as we have in this case the strong long range Coulomb force. We use the same field parameters: $\omega =0.03$ a.u., $E_0=0.07$ as above. As in the case of the short range interaction, we proceed by calculating the function defined in Eq. (14) to find the values of *t* which could be used for calculating lateral velocity distributions for the times inside the laser pulse duration. This function is shown in Fig. 4a. For the Coulomb potential the region in the $(t,v_{\perp })$-plane where $G(t,v_{\perp })$ is small, proves larger than in the case of the Yukawa potential, allowing continuous scan of the velocity distribution for all times *t* after the midpoint of the pulse.

**Figure 5:**
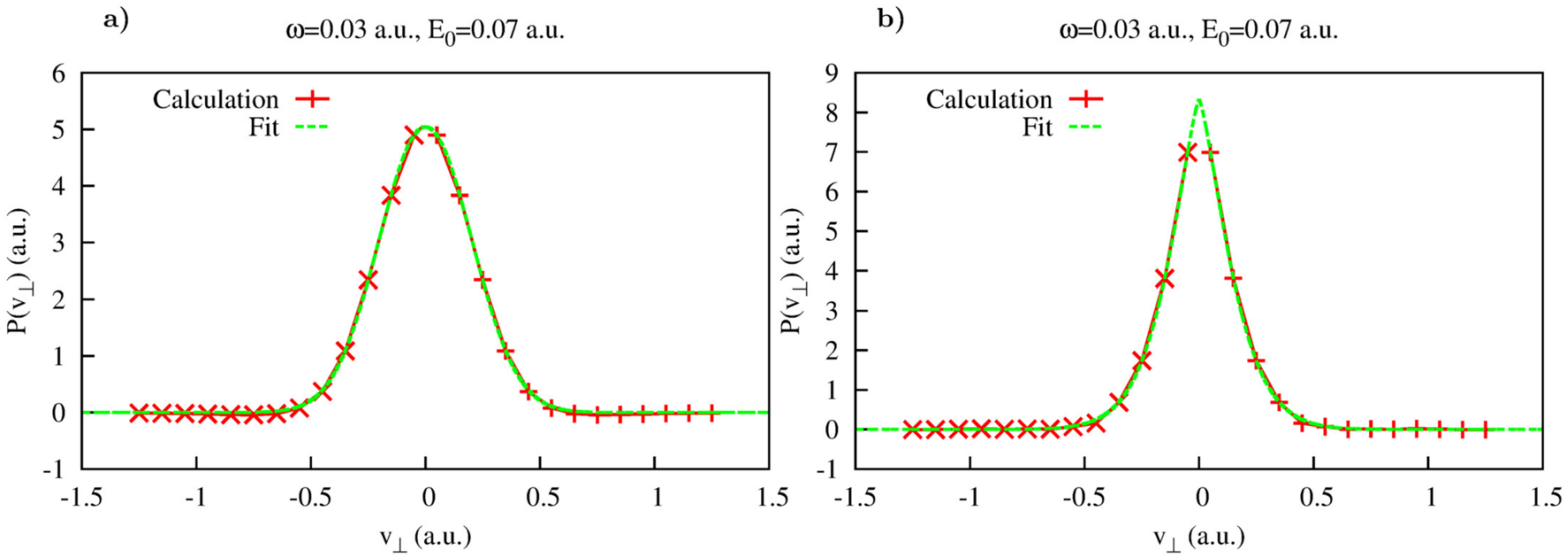
Coulomb potential. (**a**): Results of the fit of distributions computed according to Eq. (16) using Eq. (18) as a fitting anzats. (a): $t=0.548T$. (**b**): $t=T$.

**Figure 6:**
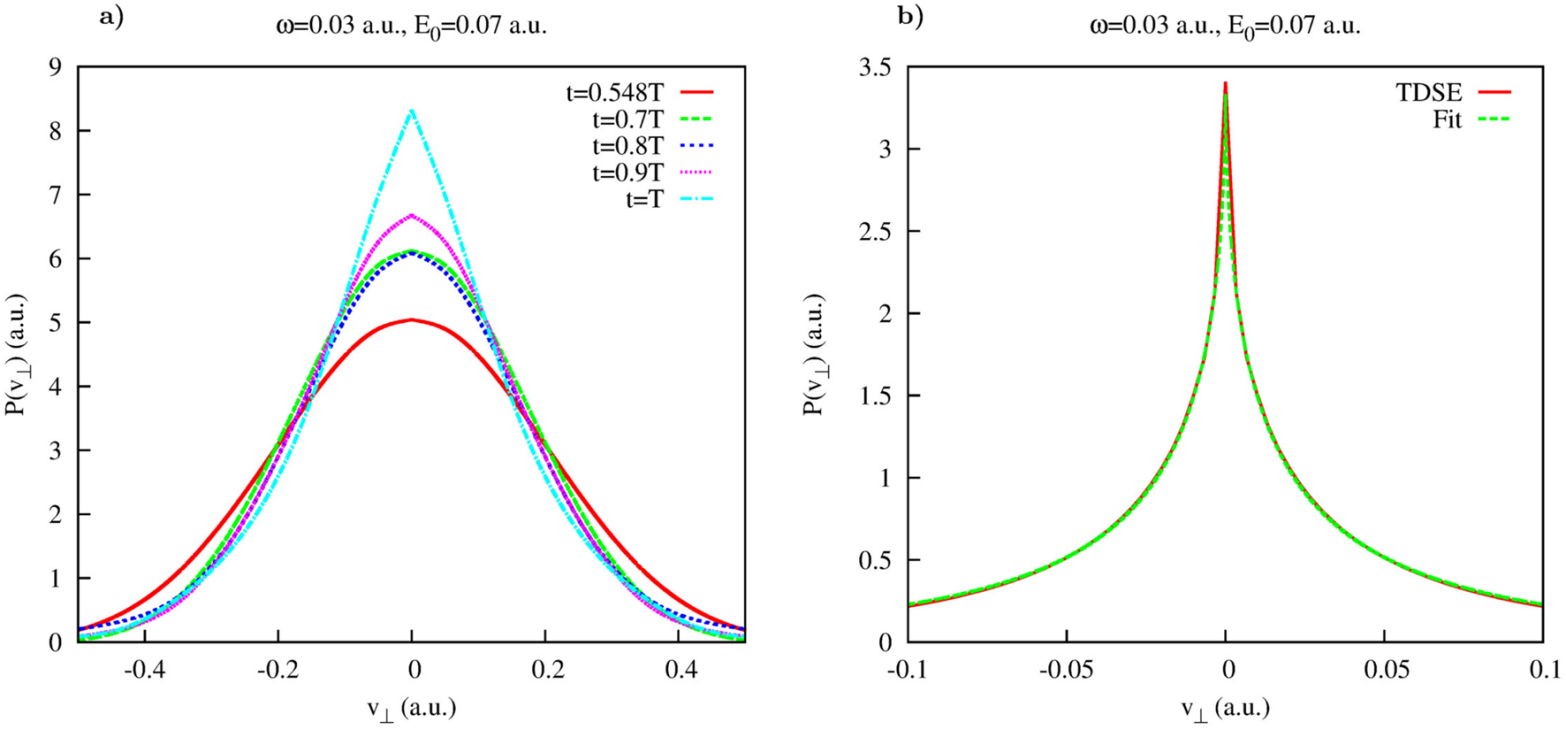
Coulomb potential. (**a**): Lateral distributions obtained using Eq. (16) for different moments of time inside the pulse duration (also shown as white dash lines in Fig. 4). (**b**): Asymptotic distribution obtained using Eq. (19).

The distribution $P(v_{\perp },t)$ is shown in Fig. 4b, and the cuts $P(v_{\perp },t_k)$ taken along the lines $t=0.548 T$ and $t=T$ are shown in Fig. 5. We also show results of the fitting procedure applied to the calculated distributions. The fitting procedure that we employ is based on the expression (18) which is non-analytical at $v_{\perp }=0$, thereby taking into account presence of a cusp^48^ for the Coulomb case^48^.

**Figure 7:**
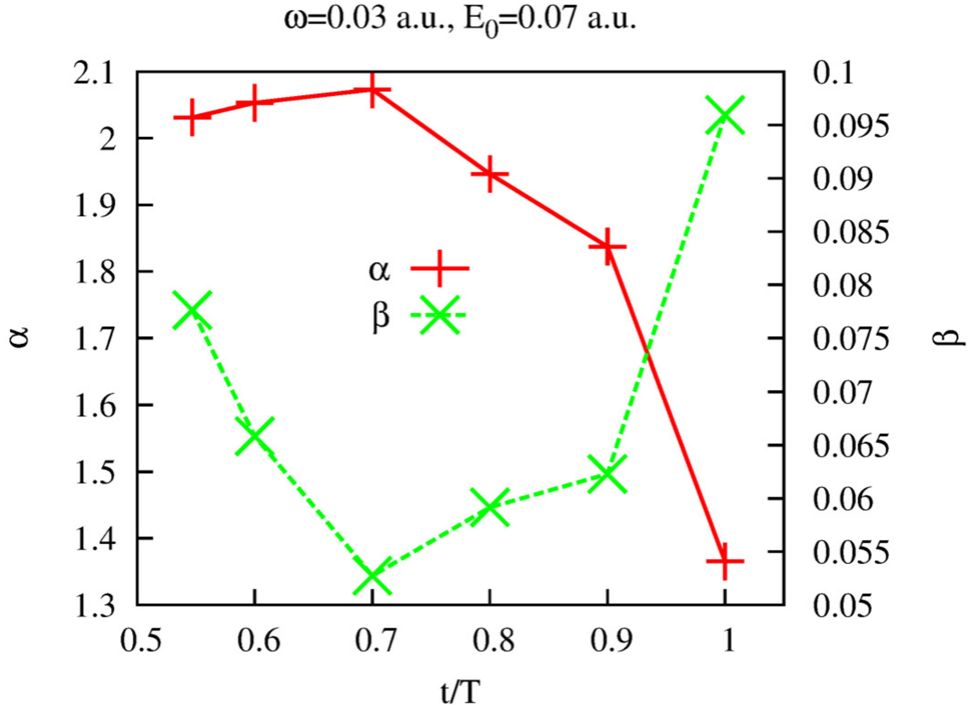
Coulomb potential. Parameters $\alpha$ and $\beta$ of the fit based on Eq. (18) as functions of time inside the interval of the laser pulse duration.

As one can see, the fits based on (18) reproduce fairly well the calculated lateral velocity distributions. One can also note that the distribution is more sharply peaked at the end of the pulse than at the moment $t=0.548T$ near the midpoint of the pulse. This evolution of the lateral velocity distribution is presented in more detail in Fig. 6 for different moments of time inside the laser pulse duration. One can observe from Fig. 6a that the non-analytical character of the velocity distribution and its departure from the Gaussian become progressively more pronounced as we approach the end of the laser pulse. This evolution of the lateral velocity distribution does not stop there, of course, long range Coulomb force keeps distorting the velocity distribution long after the pulse is gone. The asymptotic lateral velocity distribution obtained using Eq. (19) is shown in Fig. 6b. One can see that it is much sharper yet than the distribution we obtain for $T=1$. This evolution of the later velocity distributions we see in the Coulomb case agrees with the observation made in^50^, where it was noted that the cusp formation requires large (strictly speaking infinite) time. At any finite moment of time, the lateral velocity distribution remains an analytical function of $v_{\perp }$ which however, becomes, as time progresses, increasingly sharper in a vicinity of the point $v_{\perp }=0$^50^, approaching thus the cusp-like behavior present in the asymptotic (i.e. obtained for the infinite time) distribution shown in Fig. 6b. This process of the cusp formation is illustrated in Fig. 7, where we show evolution of the fitting parameters in Eq. (18) with time. One can see that parameter $\alpha$ in Eq. (18) whose role in this expression is to mimic the cusp-like behavior, progressively decreases from the nearly Gaussian value $\alpha \approx 2$, at times close to the instant of ionization, to the value $\alpha \approx 1.35$ at $t=T$. Such a low value of $\alpha$ is responsible for the rather sharp character of the lateral velocity distribution we observe for $t=T$ in Fig. 6a. Indeed, the value $\alpha \approx 1.35$ at $t=T$ implies that the first derivative of the lateral velocity distribution at $v_{\perp }=0$ is continuous and finite, while the second derivative is infinite. The asymptotic infinite time distribution shown in Fig. 6b is sharper yet, we obtain the value $\alpha =0.55$ if we apply the fit based on Eq. (18) to the distribution we calculate using Eq. (19). That means that for the asymptotic distribution it is the first derivative which is infinite at $v_{\perp }=0$. These observations illustrate again the statement made in^50^ to which we referred above, the cusp in the lateral velocity distribution becomes fully formed in the limit of infinite time.

### Field dependence of the lateral velocity distributions

We present in this section the results we obtain for the lateral velocity distribution for different driving pulse parameters. The aim of performing these calculations was primarily to convince ourselves that the procedure we devised for the calculation of the lateral velocity distributions can be applied for a wide range of the laser field parameters. For the calculations presented below we use the frequency $\omega =0.057$ a.u., and we vary the peak electric field strength. We follow the same strategy we used above and begin with presenting the analysis of the function $G(t,v_{\perp })$ defined in Eq. (14).

**Figure 8:**
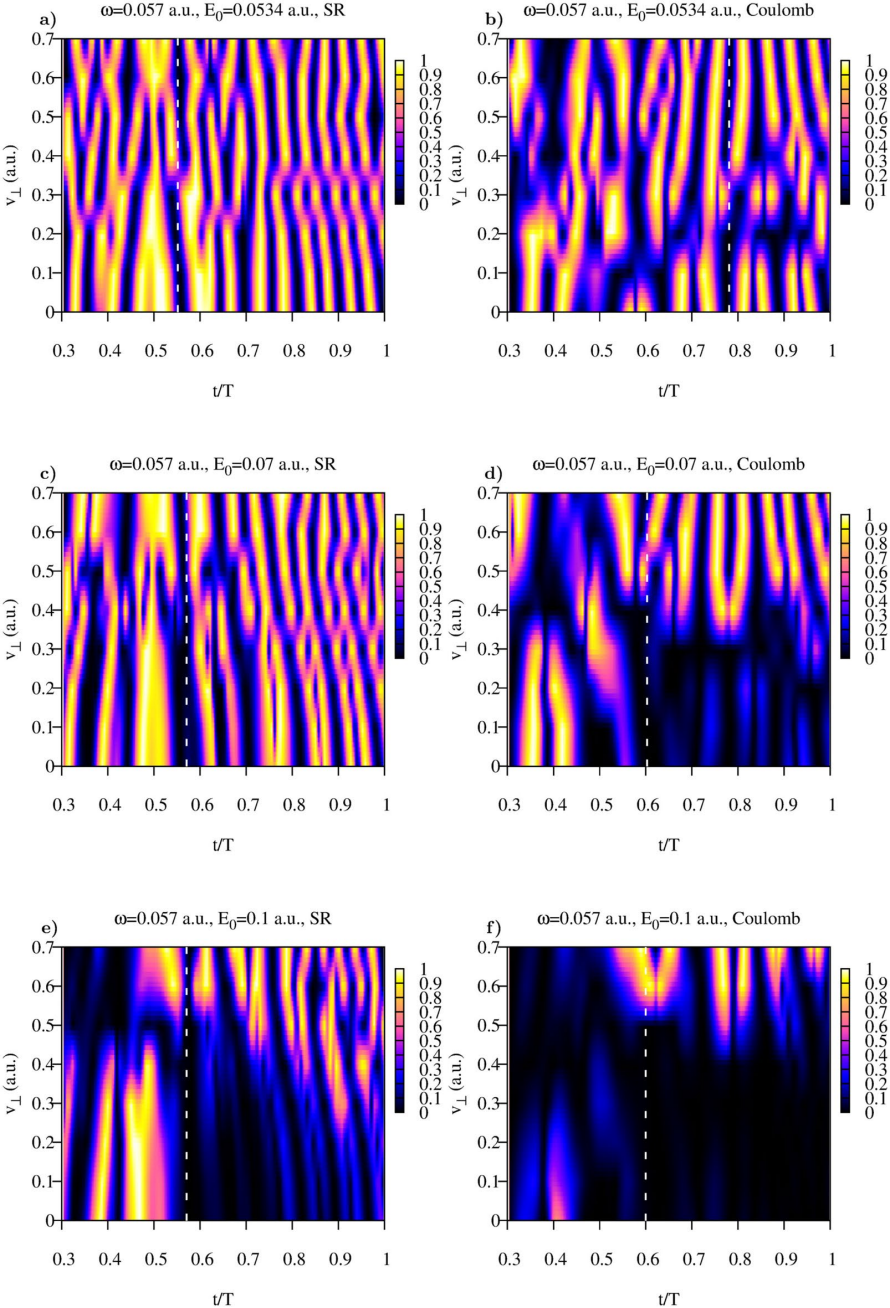
Coulomb and Yukawa potentials. Function $G(v_{\perp },t)$ for different field strengths.

This function is shown in Fig. 8 for both Yukawa and Coulomb potentials. Choosing the values of *t* where the function $G(t,v_{\perp })$ is small for the wide enough intervals of $v_{\perp }$ we can, as we did above, calculate the distributions defined in Eq. (16) for the range of the lateral velocities in which the distribution is predominantly concentrated.

**Figure 9:**
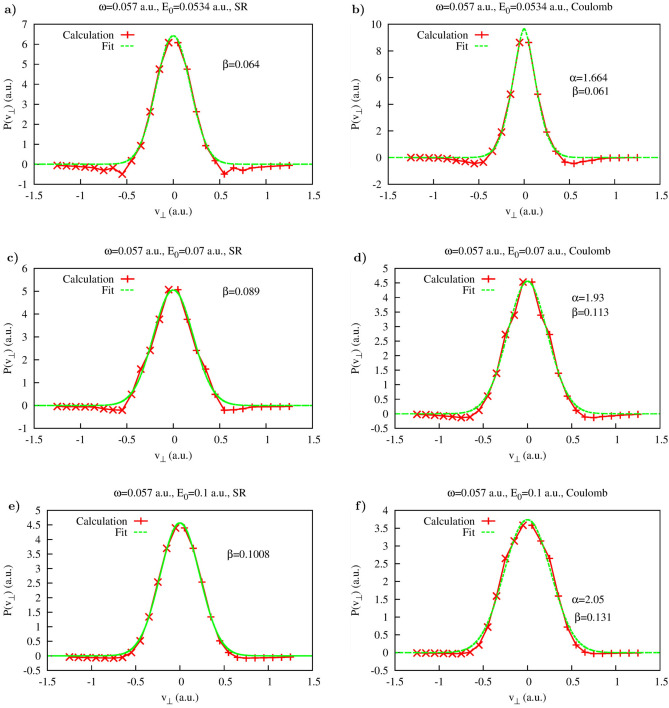
Coulomb and Yukawa potentials. Lateral distributions obtained using Eq. (16) for the following moments of time inside the pulse duration (also shown as white dash lines in Fig. 8). (**a**): $t=0.552T$, (**b**):$t=0.781T$, (**c**):$t=0.571T$, (**d**): $t=0.603T$, (**e**):$t=0.571T$, (**f**): $t=0.609T$.

**Figure 10:**
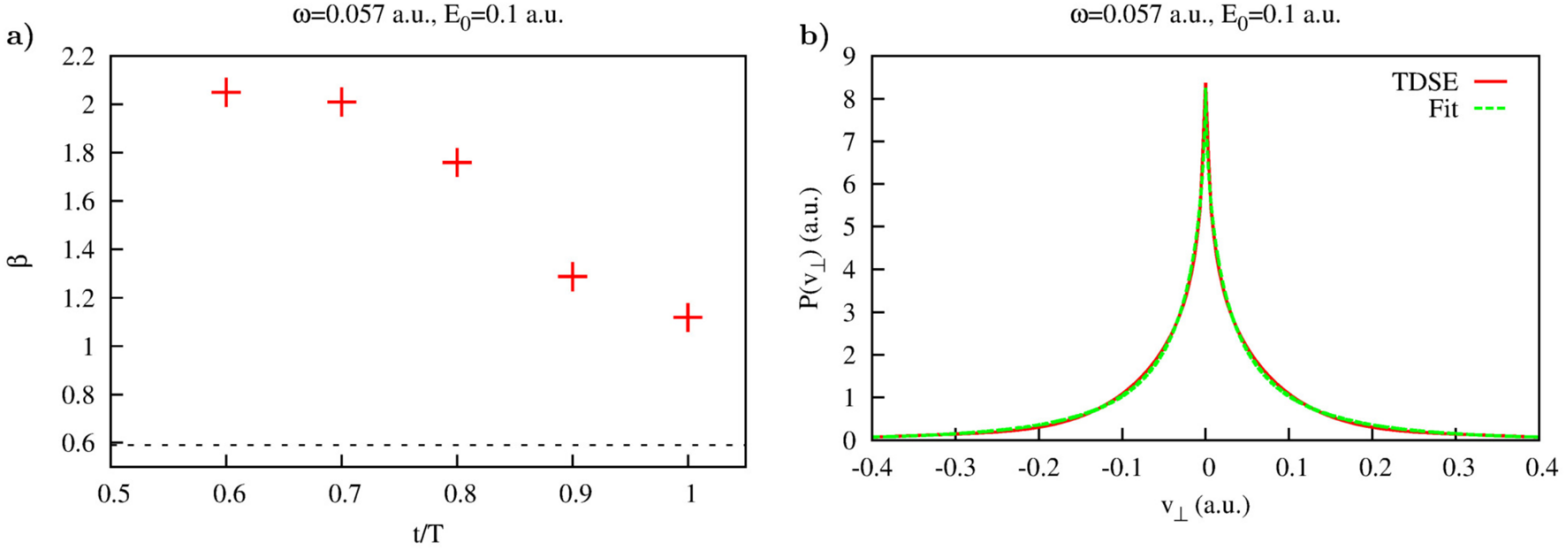
Coulomb potential. (**a**): Parameter $\beta$ of the fit based on Eq. (18) as function of time inside the interval of the laser pulse duration. (**b**): Asymptotic lateral velocity distribution obtained using Eq. (19).

**Figure 11:**
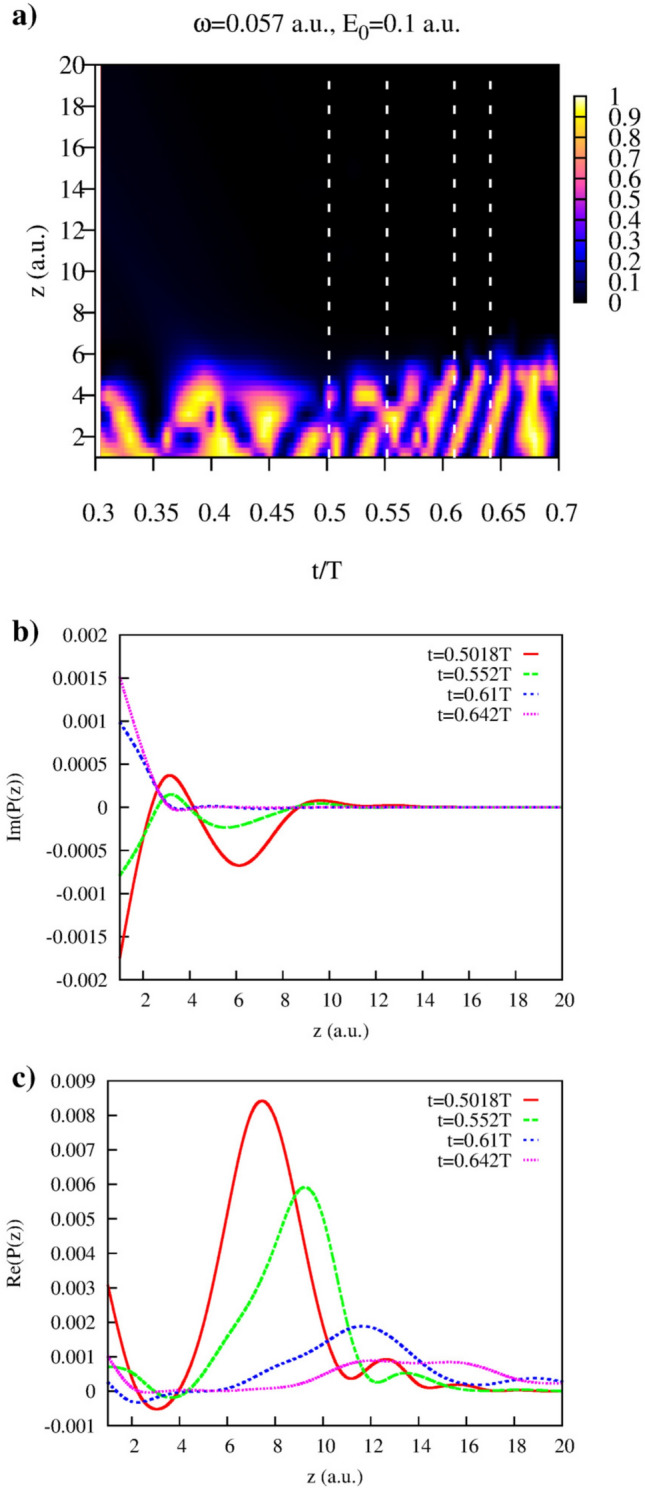
Yukawa potential. (**a**): Function *G*(*z*, *t*). Dotted white lines show the cuts at time-values $t=0.5018T$, $t=0.552T$, $t=0.61T$, and $t=0.642T$ for which the imaginary (**b**), and real (**c**) parts of the joint distributions *P*(*z*) were calculated.

The results of these calculations of the lateral velocity distributions for different driving field strengths are shown in Fig. 9. Also shown are the results of the fitting procedures based on Eqs. (17) and (18) for the Yukawa and Coulomb potentials, respectively. The fitting expressions (17) and (18) reproduce calculated distributions fairly well. The values of the fitting parameters we obtain are indicated in Fig. 9. For the short range Yukawa potential the values of the fitting parameter $\beta$ are of primary interest. As one can see, these values indeed satisfy the relation $\beta \approx E_0$, which one would expect basing on the SFA formula (15). We see again that in the case of the short range interaction, the shapes and widths of the lateral distributions we obtain for the time moments inside the laser pulse duration, coincide with the asymptotic distribution (15), describing velocity distribution at the detector. This agrees fully with the SFA-based scenario we mentioned above, according to which the lateral velocity distribution does not evolve with time once electron is ionized because of the short range character of the atomic potential assumed in the standard form of the SFA. For the Coulomb potential the fitting parameters are less informative. As we mentioned above, the long range Coulomb forces exert considerable effect on the lateral velocity distributions so that the system must evolve for a long time for the distribution to approach its limiting asymptotic form. We illustrate the character of this approach for the driving laser frequency $\omega =0.057$ a.u. in Fig. 10a, where we show time-evolution of the fitting parameter $\beta$ in Eq. (18). The asymptotic distribution (19) and its fit (18) are shown in Fig. 10b. One can see that after the ionization event $\beta$ starts decreasing, so that the lateral velocity distribution becomes progressively sharper at $v_{\perp }=0$. At the end of the pulse $\beta \approx 1$, which is still far from the value $\beta \approx 0.59$ which our fitting procedure (18) gives for the asymptotic distribution shown in Fig. 10b. We see thus the same behavior of the cusp formation that we saw in Fig. 7 for the driving laser frequency $\omega =0.03$ a.u. The cusp is not fully formed at the end of the pulse, the parameter characterizing the sharpness of the cusp needs more time yet to reach its asymptotic value.

## Conclusion

We presented a study of the lateral velocity distribution and its development in time during the interval of the laser pulse duration. Our approach is based on the application of the notion of the joint probability. Though this notion is not very well defined in QM and may sometimes lead to unphysical (e.g. complex) probability distributions, one may find situations when such a joint probability becomes a meaningful concept. This opens a way to tackle the questions which are somewhat difficult to address, in particular, to study the characteristics of the ionized electron for times inside the interval of the laser pulse duration. As we mentioned above, for the moments of time inside the interval of the laser pulse duration, when the wave-packet describing ionized electron is not fully formed and is still partly inside the atom, it is not easy to unambiguously single out the part of the wave-function describing ionized electron from the total wave-function of the system.

In the framework of our approach this ambiguity is resolved by calculating joint probability of two events, event *A* being detection of the lateral electron’s velocity in a given volume of the electron’s velocity space and event *B* being electron’s detection by a detector placed far away from the atom. We saw, that though the joint probability thus defined cannot be used for all moments of time inside the laser pulse duration, since operators corresponding to the observables *A* and *B* generally do not commute, one can find the instances when these operators commute approximately, allowing us to define sensible joint probability distribution of the events *A* and *B*.

By following this strategy we were able to track the time-evolution of the lateral velocity distributions on the time interval between the moment of ionization and the end of the laser pulse. We saw that in the case of the short range Yukawa potential, this evolution reproduces the standard SFA scenario. The shape of the lateral velocity distributions for times inside the laser pulse is reproduced pretty accurately by the SFA formula (15), and it changes very little after the moment of ionization. This is, of course, a consequence of the short range character of the Yukawa potential, which has little influence of the motion of the ionized electron while electric field alone cannot alter velocities in lateral directions. We observe quite different behavior in the case of the Coulomb potential, which is expected since long range Coulomb force can, strictly speaking, never be neglected. Since the average of the electric field over an optical cycle is zero, the long range Coulomb force may affect electron’s motion to a considerable degree even when electron is far away from the ionic core. This effects manifests itself, in particular, in the process of the time-development of the cusp in the lateral velocity distributions in the Coulomb case. We saw that the lateral velocity distributions we compute using Eq. (16) become progressively more sharply peaked near the point $v_{\perp }=0$, as illustrated in Figs. 7 and 10, thus showing the development of a cusp. Nevertheless, even at the end of the pulse this development is far from being complete, the values of the parameter $\beta$ characterizing ”sharpness” of the cusp are still far from the value we retrieve from the asymptotic (infinite time) distribution (19).

In the present work we used as objects of study the simple one-electron targets, the hydrogen atom and its counterpart described by the short-range Yukawa potential with the same ionization potential, in the field of a linearly polarized electromagnetic wave. The theoretical framework we used can be applied for more complicated targets as well, such as heavier atomic species or multiply charge ions with larger ionization potential. We would expect qualitatively similar results for these targets. In the absence of the long range Coulomb force the formation of the lateral velocity distributions, as we saw, is very well captured by the SFA, which does not rely on any information about atomic target at all, except the value of the ionization potential. We believe, therefore, that for the targets with higher ionization potential, described by the short range forces, we should obtain results similar to the results we presented above for the Yukawa SR potential, with the shape of the lateral velocity distribution reproduced fairly well for times inside the laser pulse by the SFA formula (15) and changing insignificantly after the moment of ionization. For the targets with the ionic potential exhibiting long range Coulomb tail (e.g. the multicharged ions) we would expect stronger yet effect of the Coulomb field on the cusp formation. That should give us the overall picture of the cusp formation qualitatively similar to the case of hydrogen we studied above, resulting in a considerable evolution of the cusp parameter in the lateral velocity distribution, like the one shown in Fig. 10. For the systems with stronger Coulomb forces the particular values of the parameters characterizing the cusp can, of course, be completely different from those shown in Fig. 10.

We can apply this technique to other field geometries as well, in particular to the case of the circularly or elliptically polarized electromagnetic fields. The case of the driving field polarization different from the linear one is of particular interest since for elliptically polarized field the tunneling electronic wave packet possesses an initial transverse momentum due to the non-adiabatic effects^51,52^. Calculations for such field geometry are considerably more time consuming because of the necessity of performing multiple solutions of the TDSE for the much more computationally demanding case of the non-linear polarization. We plan, nevertheless, to perform such calculations in the future to study these highly interesting non-adiabatic effects.

We also note that our approach is by no means limited to the study of the lateral velocity distributions only. By choosing other observables to represent event *A* at times inside the interval of the laser pulse duration and applying the strategy we described above, we can study evolution of the corresponding distributions. As an illustration of this statement we consider briefly some results we obtain for another choice of the event *A*. Let us define *A* as an event consisting in finding the electron in a region around the point ${{\varvec{r}}}_0$ in space at the moment of time *t*. More specifically, we define the projection operator $\hat{Q}_{{{\varvec{r}}}_0}$ representing this observable as: $\hat{Q}_{{{\varvec{r}}}_0} = |\phi _{{{\varvec{r}}}_0}({{\varvec{r}}})\rangle \langle \phi _{{{\varvec{r}}}_0}({{\varvec{r}}})|$. For $|\phi _{{{\varvec{r}}}_0}({{\varvec{r}}})\rangle$ we use the Gaussian form: $\displaystyle \phi _{{{\varvec{r}}}_0}({{\varvec{r}}}) = N e^{-a({{\varvec{r}}}-{{\varvec{r}}}_0)^2)}$, where *N* is the normalization factor and we use the value $a=2\ln 2$ for the parameter *a*. This parameter defines the ’resolution’ with which we can scrutinize the coordinate space, and it is approximately one atomic unit of length for the choice of the parameter *a* we made. In the Eq. (13) we can replace now the projection operator $\hat{Q}_i$ with the projection operator $\hat{Q}_{{{\varvec{r}}}_0}$, and the resulting expression will give the joint probability $P({{\varvec{r}}}_0)$ of finding electron ionized after the end of the pulse and finding it in a location nearby ${{\varvec{r}}}_0$ at the moment *t*. In other words, the joint probability thus calculated gives us a distribution of the ionized electron coordinates at times inside the laser pulse. We proceed with the calculations in exactly the same way as we did before, obtaining the results shown in Fig. 11 for the case of the short range Yukawa potential. We use the same pulse parameters as above and report the results for the peak field strengths $E_0=0.1$ a.u. To simplify the notation we drop the subscripts and we will write simply ${{\varvec{r}}}$ instead of ${{\varvec{r}}}_0$ in the formulas below which should not cause confusion.

We show in Fig. 11a the function *G*(*z*, *t*) calculated according to Eq. (14), with projection operator $\hat{Q}_k$ replaced with the operator $\hat{Q}_{{{\varvec{r}}}}$ for ${{\varvec{r}}}=(0,0,z)$. We remind that in our geometry *z*-direction is the polarization direction, so by choosing this direction in space we can study the coordinate distribution for the ionized electron along the laser polarization direction. One can see from Fig. 11a that for sufficiently large *z* and all *t* *G*(*z*, *t*) is small. Joint distributions computed according to the Eq. (13) give us, therefore, reliable distributions for the ionized electron $z-$ coordinate for all *t* and at least some interval of *z*-values. This can also be seen from Fig. 11b,c where we show imaginary and real parts of the joint distributions *P*(*z*) computed according to Eq. (13). One can see that for the values of *z* of interest (near the peaks of the real parts of *P*(*z*)) imaginary part of *P*(*z*) is at least an order of magnitude smaller than the real part. We obtain thus the meaningful ionized electron’s coordinate distributions for the most important and interesting intervals of *z*, where the bulk of a distribution is concentrated. As one can see from Fig. 11c electron ionized at the moment near the field maximum at $t=0.5T$ is located at $z\approx 7$ a.u. This is not very different from a simple estimate for the tunnel exit location based on the energy conservation formula for an electron moving in the combined field of the ionic core and the static electric field with the amplitude $E_0$ (the so-called Field Direction Model (FDM)^18^), which, for the Yukawa potential we consider and $E_0=0.1$ a.u. is $z\approx 5$ a.u.

Using this approach one can obtain information about other nonlinear phenomena as well. The rescattering process, for instance, is at the core of the HHG process and is responsible for the formation of the high energy part of the ATI spectra^11^. Consider, for instance, the ATI process. By choosing event *B* to be detection of the electron in the ionized state and event *A* to be finding the electron’s coordinate in a certain region of space (as we did above studying the ionized electron’s coordinate distributions), we can single out the part of the wave-function describing the ionized wave-packet, and study rescattering process in detail by following spatial and temporal evolution of the ionized wave-packet.

## Data Availability

All relevant data are available from the authors upon request sent to I.A.I.
